# Supplementary material for: Modeling glioblastoma heterogeneity as a dynamic network of cell states
Source: Mol Syst Biol. 2021 Sep 16;17(9):e10105. doi: 10.15252/msb.202010105 (PMC8444284; doi:10.15252/msb.202010105)
Supplement: Supplementary file 6 — Source Data for Figure 5 [file MSB-17-e10105-s004.zip › Figure5A_sourcedata/GSEA_3017/hallmarks_stateD.GseaPreranked.1621934542601/HALLMARK_E2F_TARGETS.html]

Details for gene set HALLMARK\_E2F\_TARGETS[GSEA]

|  || Dataset | state23017 |
| Phenotype | NoPhenotypeAvailable |
| Upregulated in class | na\_neg |
| GeneSet | HALLMARK\_E2F\_TARGETS |
| Enrichment Score (ES) | -0.40654048 |
| Normalized Enrichment Score (NES) | -2.1747713 |
| Nominal p-value | 0.0 |
| FDR q-value | 0.008626049 |
| FWER p-Value | 0.008 |
Table: GSEA Results Summary

  

Fig 1: Enrichment plot: HALLMARK\_E2F\_TARGETS      
 Profile of the Running ES Score & Positions of GeneSet Members on the Rank Ordered List

  

| PROBE | GENE SYMBOL | GENE\_TITLE | RANK IN GENE LIST | RANK METRIC SCORE | RUNNING ES | CORE ENRICHMENT || 1 | PTTG1 |  |  | 14 | 0.490 | -0.0010 | No |
| 2 | MYC |  |  | 77 | 0.313 | -0.0452 | No |
| 3 | HMGB3 |  |  | 80 | 0.311 | -0.0403 | No |
| 4 | BIRC5 |  |  | 130 | 0.271 | -0.0747 | No |
| 5 | PPM1D |  |  | 162 | -0.251 | -0.0948 | No |
| 6 | NUP205 |  |  | 165 | -0.251 | -0.0911 | No |
| 7 | RFC1 |  |  | 190 | -0.253 | -0.1054 | No |
| 8 | MKI67 |  |  | 206 | -0.254 | -0.1123 | No |
| 9 | MCM2 |  |  | 226 | -0.256 | -0.1224 | No |
| 10 | TRIP13 |  |  | 294 | -0.263 | -0.1718 | No |
| 11 | POLA2 |  |  | 312 | -0.264 | -0.1801 | No |
| 12 | HMMR |  |  | 319 | -0.266 | -0.1794 | No |
| 13 | EXOSC8 |  |  | 347 | -0.268 | -0.1958 | No |
| 14 | TUBB |  |  | 410 | -0.274 | -0.2408 | No |
| 15 | CSE1L |  |  | 421 | -0.276 | -0.2432 | No |
| 16 | NUP107 |  |  | 422 | -0.276 | -0.2373 | No |
| 17 | CDC25B |  |  | 472 | -0.282 | -0.2715 | No |
| 18 | EED |  |  | 569 | -0.293 | -0.3440 | No |
| 19 | SLBP |  |  | 573 | -0.295 | -0.3402 | No |
| 20 | DSCC1 |  |  | 596 | -0.299 | -0.3518 | No |
| 21 | SPAG5 |  |  | 605 | -0.301 | -0.3520 | No |
| 22 | RFC2 |  |  | 607 | -0.302 | -0.3464 | No |
| 23 | MCM3 |  |  | 625 | -0.305 | -0.3538 | No |
| 24 | DCK |  |  | 631 | -0.307 | -0.3514 | No |
| 25 | DNMT1 |  |  | 659 | -0.312 | -0.3669 | No |
| 26 | RAD51C |  |  | 670 | -0.313 | -0.3684 | No |
| 27 | RAD21 |  |  | 674 | -0.314 | -0.3642 | No |
| 28 | TMPO |  |  | 688 | -0.317 | -0.3681 | No |
| 29 | PLK4 |  |  | 699 | -0.319 | -0.3695 | No |
| 30 | KIF4A |  |  | 706 | -0.320 | -0.3676 | No |
| 31 | SPC24 |  |  | 747 | -0.330 | -0.3934 | No |
| 32 | PRIM2 |  |  | 764 | -0.336 | -0.3994 | Yes |
| 33 | UBR7 |  |  | 767 | -0.336 | -0.3939 | Yes |
| 34 | GINS1 |  |  | 780 | -0.338 | -0.3965 | Yes |
| 35 | MCM4 |  |  | 783 | -0.339 | -0.3909 | Yes |
| 36 | CDC20 |  |  | 793 | -0.341 | -0.3911 | Yes |
| 37 | BRCA2 |  |  | 808 | -0.347 | -0.3952 | Yes |
| 38 | NCAPD2 |  |  | 811 | -0.347 | -0.3894 | Yes |
| 39 | CENPM |  |  | 818 | -0.349 | -0.3869 | Yes |
| 40 | MAD2L1 |  |  | 819 | -0.350 | -0.3794 | Yes |
| 41 | GINS4 |  |  | 824 | -0.351 | -0.3753 | Yes |
| 42 | CBX5 |  |  | 842 | -0.356 | -0.3816 | Yes |
| 43 | MRE11 |  |  | 847 | -0.356 | -0.3773 | Yes |
| 44 | CCNE1 |  |  | 850 | -0.357 | -0.3713 | Yes |
| 45 | RFC3 |  |  | 852 | -0.358 | -0.3646 | Yes |
| 46 | CDKN1B |  |  | 862 | -0.359 | -0.3643 | Yes |
| 47 | PRKDC |  |  | 871 | -0.363 | -0.3631 | Yes |
| 48 | SMC1A |  |  | 885 | -0.367 | -0.3659 | Yes |
| 49 | TCF19 |  |  | 890 | -0.369 | -0.3614 | Yes |
| 50 | TK1 |  |  | 902 | -0.375 | -0.3624 | Yes |
| 51 | TUBG1 |  |  | 917 | -0.381 | -0.3658 | Yes |
| 52 | MSH2 |  |  | 927 | -0.385 | -0.3650 | Yes |
| 53 | USP1 |  |  | 930 | -0.386 | -0.3584 | Yes |
| 54 | PSIP1 |  |  | 934 | -0.388 | -0.3526 | Yes |
| 55 | KIF18B |  |  | 935 | -0.388 | -0.3443 | Yes |
| 56 | DIAPH3 |  |  | 943 | -0.390 | -0.3418 | Yes |
| 57 | RPA1 |  |  | 946 | -0.393 | -0.3351 | Yes |
| 58 | XPO1 |  |  | 967 | -0.400 | -0.3429 | Yes |
| 59 | POLD3 |  |  | 1002 | -0.415 | -0.3620 | Yes |
| 60 | RBBP7 |  |  | 1006 | -0.418 | -0.3556 | Yes |
| 61 | CCP110 |  |  | 1010 | -0.420 | -0.3491 | Yes |
| 62 | TIMELESS |  |  | 1024 | -0.429 | -0.3506 | Yes |
| 63 | LIG1 |  |  | 1035 | -0.437 | -0.3495 | Yes |
| 64 | KIF22 |  |  | 1051 | -0.446 | -0.3523 | Yes |
| 65 | CDKN2C |  |  | 1067 | -0.454 | -0.3549 | Yes |
| 66 | EZH2 |  |  | 1070 | -0.455 | -0.3469 | Yes |
| 67 | MMS22L |  |  | 1072 | -0.457 | -0.3380 | Yes |
| 68 | DEPDC1 |  |  | 1074 | -0.457 | -0.3291 | Yes |
| 69 | PSMC3IP |  |  | 1075 | -0.457 | -0.3193 | Yes |
| 70 | PLK1 |  |  | 1082 | -0.460 | -0.3145 | Yes |
| 71 | BUB1B |  |  | 1111 | -0.477 | -0.3273 | Yes |
| 72 | RACGAP1 |  |  | 1112 | -0.479 | -0.3171 | Yes |
| 73 | E2F8 |  |  | 1116 | -0.485 | -0.3092 | Yes |
| 74 | BARD1 |  |  | 1117 | -0.486 | -0.2989 | Yes |
| 75 | ASF1B |  |  | 1133 | -0.498 | -0.3006 | Yes |
| 76 | PCNA |  |  | 1142 | -0.504 | -0.2964 | Yes |
| 77 | MXD3 |  |  | 1153 | -0.514 | -0.2936 | Yes |
| 78 | TFRC |  |  | 1159 | -0.521 | -0.2866 | Yes |
| 79 | BRCA1 |  |  | 1171 | -0.541 | -0.2842 | Yes |
| 80 | ORC6 |  |  | 1179 | -0.551 | -0.2782 | Yes |
| 81 | KIF2C |  |  | 1183 | -0.558 | -0.2687 | Yes |
| 82 | CDCA3 |  |  | 1187 | -0.563 | -0.2592 | Yes |
| 83 | CKS1B |  |  | 1198 | -0.577 | -0.2551 | Yes |
| 84 | DUT |  |  | 1223 | -0.633 | -0.2614 | Yes |
| 85 | SMC4 |  |  | 1224 | -0.638 | -0.2478 | Yes |
| 86 | MYBL2 |  |  | 1225 | -0.639 | -0.2342 | Yes |
| 87 | CKS2 |  |  | 1226 | -0.641 | -0.2205 | Yes |
| 88 | CDCA8 |  |  | 1239 | -0.669 | -0.2161 | Yes |
| 89 | AURKB |  |  | 1250 | -0.700 | -0.2094 | Yes |
| 90 | MELK |  |  | 1251 | -0.701 | -0.1945 | Yes |
| 91 | UBE2T |  |  | 1256 | -0.719 | -0.1825 | Yes |
| 92 | HMGB2 |  |  | 1263 | -0.736 | -0.1717 | Yes |
| 93 | RAD51AP1 |  |  | 1264 | -0.741 | -0.1559 | Yes |
| 94 | TACC3 |  |  | 1268 | -0.751 | -0.1424 | Yes |
| 95 | AURKA |  |  | 1271 | -0.763 | -0.1278 | Yes |
| 96 | ATAD2 |  |  | 1275 | -0.775 | -0.1137 | Yes |
| 97 | SPC25 |  |  | 1294 | -0.932 | -0.1087 | Yes |
| 98 | RRM2 |  |  | 1295 | -0.933 | -0.0888 | Yes |
| 99 | KPNA2 |  |  | 1297 | -0.972 | -0.0689 | Yes |
| 100 | CDKN1A |  |  | 1306 | -1.046 | -0.0532 | Yes |
| 101 | CDK1 |  |  | 1311 | -1.433 | -0.0260 | Yes |
| 102 | TOP2A |  |  | 1313 | -1.528 | 0.0057 | Yes |
Table: GSEA details [plain text format]

  

Fig 2: HALLMARK\_E2F\_TARGETS: Random ES distribution      
 Gene set null distribution of ES for **HALLMARK\_E2F\_TARGETS**
